# The changing epidemiology of pulmonary infection in children and adolescents with cystic fibrosis: an 18-year experience

**DOI:** 10.1038/s41598-024-59658-4

**Published:** 2024-04-20

**Authors:** Jagdev Singh, Sharon Hunt, Sharon Simonds, Christie Boyton, Anna Middleton, Matthew Elias, Susan Towns, Chetan Pandit, Paul Robinson, Dominic A. Fitzgerald, Hiran Selvadurai

**Affiliations:** 1 Department of Respiratory Medicine, The Children’s Hospital at Westmead, Sydney, NSW Australia; 2 Department of Pharmacy, The Children’s Hospital at Westmead, Sydney, NSW Australia; 3 Discipline of Child and Adolescent Health, Sydney Medical School, University of Sydney, Sydney, NSW Australia

**Keywords:** Cystic fibrosis, Bacterial infection

## Abstract

The impact of evolving treatment regimens, airway clearance strategies, and antibiotic combinations on the incidence and prevalence of respiratory infection in cystic fibrosis (CF) in children and adolescents remains unclear. The incidence, prevalence, and prescription trends from 2002 to 2019 with 18,339 airway samples were analysed. *Staphylococcus aureus* [− 3.86% (95% CI − 5.28–2.43)] showed the largest annual decline in incidence, followed by *Haemophilus influenzae* [− 3.46% (95% CI − 4.95–1.96)] and *Pseudomonas aeruginosa* [− 2.80%95% CI (− 4.26–1.34)]. *Non-tuberculous mycobacteria* and *Burkholderia cepacia* showed a non-significant increase in incidence. A similar pattern of change in prevalence was observed. No change in trend was observed in infants < 2 years of age. The mean age of the first isolation of *S. aureus* (*p* < 0.001), *P. aeruginosa* (*p* < 0.001), *H. influenza* (*p* < 0.001), *Serratia marcescens* (*p* = 0.006) and *Aspergillus fumigatus* (*p* = 0.02) have increased. Nebulised amikacin (+ 3.09 ± 2.24 prescription/year, *p* = 0.003) and colistin (+ 1.95 ± 0.3 prescriptions/year, *p* = 0.032) were increasingly prescribed, while tobramycin (− 8.46 ± 4.7 prescriptions/year, *p* < 0.001) showed a decrease in prescription. Dornase alfa and hypertonic saline nebulisation prescription increased by 16.74 ± 4.1 prescriptions/year and 24 ± 4.6 prescriptions/year (*p* < 0.001). There is a shift in CF among respiratory pathogens and prescriptions which reflects the evolution of cystic fibrosis treatment strategies over time.

## Introduction

The management of pulmonary infections is critical in the care of individuals with cystic fibrosis (CF). Despite an increase in the median survival age over recent years, chronic pulmonary infection and concomitant airway inflammation leading to respiratory failure still account for 80–95% of deaths in individuals with CF^1,2^. This vicious cycle of infection and inflammation begins early in life, resulting in a decline in lung function, poorer nutrition, and structural lung abnormalities^3^.

Assessing long-term epidemiological trends in CF among children poses significant challenges, with studies often limited to registry reports, of a limited timeframe^4^, involve a small number of children and adolescents^5^, focus on specific organisms of interest^6,7^, or are derived from results obtained from bronchioalveolar sampling alone^8,9^. Furthermore, larger studies conducted before the year 2000 may not reflect recent advancements in CF treatment^10–14^, highlighting the need to evaluate any changes in the incidence and prevalence of CF bacterial pathogens to establish a reference point for future therapeutic interventions.

To this end, we conducted a study to investigate the trends in the incidence and prevalence of respiratory pathogens among children and adolescents with CF since the turn of the new millennium. By evaluating long-term longitudinal data within a clinical setting in the modern era of eradication therapy^15^, we would like to determine the changes that may have occurred in different age groups over time.

## Methodology

### Study population

Children and adolescents with CF between birth to 18 years of age who were managed within a large CF centre in Australia between January 2002 and December 2019 were included in this study. Universal newborn screening of cystic fibrosis had been well-established before the study period^16^. Data collected from their existing electronic medical record included; the microbiological culture result (method of collection, date during which sample was collected with the corresponding age of the child or adolescent), and hospital pharmacy-based medication prescription data. This study was approved by the Ethics Committee of the Sydney Children’s Hospital Network (2020/ETH00815) and was conducted based on local guidelines and regulations. Exemption from consent was obtained from, and approved by the same committee.

### Clinical routine during the study period

In our centre which encompasses a large region in New South Wales, outpatient (CF clinic) reviews occur four times a year, with infants or those who are clinically unwell reviewed on a more frequent basis. During these visits, airway samples are routinely collected regardless of the presence or absence of symptoms either through spontaneous expectoration (typically in older children), oropharyngeal suctioning performed by a trained CF nurse (typically in younger children), or via bronchoalveolar lavage (BAL). Airway samples microbiological cultures are ordered based on either BAL culture order label (samples obtained via BAL) or sputum CF culture order label (samples obtained through either spontaneously expectorated sputum or airway sample obtained from oropharyngeal suctioning).

All infants less than one year of age have been prescribed oral flucloxacillin or occasionally amoxicillin and clavulanic acid from diagnosis as part of our CF clinics’ routine *Staphylococcus aureus* prophylaxis approach for over 20 years.

In terms of the microbiological practices which has remained consistent during this study period, sputum specimens have been set up on (1) MacConkey agar for gram-negative bacteria e.g., coliforms, *Pseudomonas aeruginosa,* and *Inquilinus limosus*, (2) Anaerobically incubated chocolate agar with Bacitracin for *Haemophilus influenzae*. (3) Mannitol salt agar for *S. aureus* (4) Horse blood agar for e.g., *Streptococcus pneumoniae* and *Moraxella catarrhalis*. (5) Cepacia agar for *Burkholderia cepacia* and incubated for 7 days. (6) Non-tuberculous mycobacteria (NTM) testing is performed in an external Mycobacterium Reference Laboratory (MRL) using the automated blood culture system (BD BACTEC™) and testing occurs annually. Matrix-assisted laser desorption/ionization-time of flight (MALDI-TOF) mass spectrometry (MS) has been used since 2015 for the rapid identification of organisms.

The microbiologist's report on the results of the collected airway samples is routinely reviewed by the CF team within 5–7 days after the samples are obtained. Treatment, where applicable following discussion with the primary CF physician is then prescribed. The treatment strategy includes; admission for parenteral antibiotics, a course of oral antibiotics, and/or nebulised antibiotic treatment.

### Case definitions and stratification

Incidence was defined as the first time a respiratory pathogen of interest is isolated from the sputum of the child or adolescent with CF. Once the child or adolescent is an incident case for that particular pathogen, they were excluded from the denominator for the subsequent years.

Prevalence was defined as a child or adolescent with a respiratory pathogen isolated from their sputum in a specific year. Once the child or adolescent is a prevalent case for that particular pathogen, any further positive culture of the same pathogen isolated from the same child or adolescent was excluded for the remainder of that year.

Nine organisms of clinical interest in CF were selected for analysis. This includes; *S. aureus, P. aeruginosa, H. influenza, Aspergillus fumigatus, Serratia marcescens, NTM, B. cepacia, Achromobacter xylosoxidans*, and *Stenotrophomonas maltophilia*^17^*.*

The cohort was divided into four age groups: < 2 years, 2–5 years, 6–11 years, and > 12 years. The rationale behind this age group includes (1) biological variability in terms of differences in microbiome composition, immune system development and environmental exposure e.g. home or pre-school (2) management approaches such as methods of physiotherapy, lung function testing or the availability of medications such as dornase alfa (3) to align with existing clinical trials in CF transmembrane conductance regulator (CFTR) and CF registry reports.

In terms of medications prescribed and obtained from the hospital pharmacy, prescription of oral antimicrobials (including amoxicillin and clavulanic acid, ciprofloxacin, trimethoprim/sulfamethoxazole, flucloxacillin, and itraconazole), nebulised antimicrobials (including amikacin, colistin, and tobramycin), and other medications (including dornase-alfa, hypertonic saline nebules, and CFTR modulators and correctors) were reviewed.

### Statistical analysis

We used descriptive statistics to summarise the data, reporting organism incidence and prevalence as n (%). To assess changes over time, we calculated the annual incidence and prevalence of each organism based on individual airway samples, and used regression analysis to evaluate these measures. Based on the coefficients obtained from the regression model, the average change in incidence and prevalence was presented. Prescription trends were also analysed on an individual basis. Results are reported as % change (with 95% confidence intervals) for incidence and prevalence, and as number of prescriptions/year ± standard deviation for medications prescribed. Changes in the mean age of first organism isolation were assessed using analysis of variance. All statistical calculations were performed using the SPSS Statistic Data Editor (IBM Version 28, New York, USA, 2021). Statistical significance was defined as *p* < 0.05.

## Results

### Study population and bacterial samples

During the study period, 419 children and adolescents with CF were followed up with 206 (49.2%) born on, or after 1st January 2002. A total of 18,339 airway samples were collected during the study period with 401 (2.2%) collected via bronchioalveolar lavage, with the remaining samples obtained from expectorated sputum or oropharyngeal suction.

Out of the total airway samples that were collected, 724 (3.9%) samples met the criteria for incidence and 15,332 (83.6%) samples met the criteria for prevalence as defined in the methodology of this study were included in the analysis.

### Incidence and prevalence of respiratory pathogens

Throughout the entire study period, *S. aureus* (25.1%), *P. aeruginosa* (26.2%), and *H. influenzae* (17.9%) exhibited the highest incidence among respiratory pathogens. Together, these pathogens accounted for 70% of the overall incidence over 18 years. In contrast, *B. cepacia* (0.69%), *A. xylosoxidans* (2.1%), and NTM (3.7%) had the lowest incidence across the study period, collectively representing 6.5% of the overall incidence over 18 years (Table 1).

**Table 1:** Incidence rates of pathogens isolated according to consecutive years.

*Staphylococcus aureus*	2002	2003	2004	2005	2006	2007	2008	2009	2010	2011	2012	2013	2014	2015	2016	2017	2018	2019	Change by year (95% CI)	*p*-value
	n = 13	n = 6	n = 12	n = 20	n = 11	n = 11	n = 16	n = 11	n = 14	n = 12	n = 10	n = 5	n = 10	n = 22	n = 14	n = 13	n = 6	n = 7		
Overall	6.1	2.8	5.6	9.4	5.2	5.2	7.5	5.2	6.6	5.6	4.7	2.3	4.7	10.3	6.6	6.1	2.8	3.3	− 3.861 (− 5.284–2.439)	< 0.001
0–1 year	10.9	4.0	8.9	7.9	5.9	7.9	8.9	4.0	5.9	5.0	5.9	2.0	4.0	5.0	7.9	3.0	1.0	2.0	1.186 (− 2.63–5.001)	0.543
2–5 years	2.7	2.7	4.1	16.2	6.8	2.7	8.1	8.1	8.1	8.1	2.7	1.4	4.1	10.8	1.4	6.8	1.4	4.1	− 2.413 (− 4.689–0.137)	0.038
6–11 years	0	0	0	0	0	3.4	3.4	3.4	6.9	3.4	6.9	6.9	6.9	20.7	17.2	13.8	3.4	3.4	− 1.087 (− 2.353–0.179)	0.092
≥ 12 years	0	0	0	0	0	0	0	0	0	0	0	0	11.1	33.3	0	11.1	33.3	11.1	− 0.619 (− 1.851–0.614)	0.325

*Pseudomonas aeruginosa*	2002	2003	2004	2005	2006	2007	2008	2009	2010	2011	2012	2013	2014	2015	2016	2017	2018	2019	Change by year (95% CI)	*p*-value
	n = 6	n = 4	n = 10	n = 10	n = 12	n = 10	n = 13	n = 8	n = 11	n = 7	n = 10	n = 3	n = 19	n = 15	n = 9	n = 16	n = 14	n = 13		
Overall	3.20	2.10	5.30	5.30	6.30	5.30	6.80	4.20	5.80	3.70	5.30	1.60	10	7.90	4.70	8.40	7.40	6.80	− 2.803 (− 4.263–1.343)	< 0.001
0–1 year	2.40	7.10	14.30	14.30	14.30	7.10	7.10	9.50	0	2.40	2.40	0	4.80	7.10	0	4.80	0	2.40	0.127 (− 3.878–4.132)	0.951
2–5 years	7.10	1.40	5.70	5.70	8.60	8.60	12.90	5.70	7.10	4.30	5.70	1.40	7.10	4.30	1.40	4.30	1.40	7.10	− 2.265 (− 4.561–031)	0.053
6–11 years	0	0	0	0	0	1.70	1.70	0	10.20	5.10	8.50	3.40	15.30	10.20	10.20	13.60	15.30	5.10	− 0.459 (− 1.548–0.63)	0.409
≥ 12 years	0	0	0	0	0	0	0	0	0	0	0	0	15.80	15.80	10.50	15.80	21.10	21.10	− 0.423 (− 1.529–0.683)	0.453

*Haemophilus influenza*	2002	2003	2004	2005	2006	2007	2008	2009	2010	2011	2012	2013	2014	2015	2016	2017	2018	2019	Change by year (95% CI)	*p*-value
	n = 1	n = 1	n = 0	n = 8	n = 14	n = 7	n = 14	n = 10	n = 7	n = 8	n = 8	n = 1	n = 15	n = 11	n = 10	n = 8	n = 3	n = 4		
Overall	0.80	0.80	0	6.20	10.80	5.40	10.80	7.70	5.40	6.20	6.20	0.80	11.50	8.50	7.70	6.20	2.30	3.10	− 3.456 (− 4.951–1.961)	< 0.001
0–1 year	2.30	0	0	9.10	11.40	6.80	6.80	9.10	4.50	6.80	2.30	0	9.10	9.10	13.60	4.50	2.30	2.30	2.354 (− 1.559–6.266)	0.238
2–5 years	0	2.00	0	7.80	13.70	3.90	19.60	2.00	7.80	5.90	5.90	2.00	13.70	3.90	2.00	9.80	0	0	− 2.83 (− 5.174–0.486)	0.018
6–11 years	0	0	0	0	7.10	7.10	3.60	17.90	3.60	7.10	14.30	0	14.30	10.70	7.10	0	3.60	3.60	− 2.562 (− 3.952–1.172)	< .001
≥ 12 years	0	0	0	0	0	0	0	0	0	0	0	0	0	28.60	14.30	14.30	14.30	28.60	− 0.08 (− 1.373–1.214)	0.904

*Aspergillus fumigatus*	2002	2003	2004	2005	2006	2007	2008	2009	2010	2011	2012	2013	2014	2015	2016	2017	2018	2019	Change by year (95% CI)	*p*-value
	n = 0	n = 1	n = 1	n = 1	n = 2	n = 3	n = 8	n = 8	n = 11	n = 8	n = 2	n = 0	n = 7	n = 5	n = 13	n = 11	n = 6	n = 6		
Overall	0	1.10	1.10	1.10	2.20	3.20	8.60	8.60	11.80	8.60	2.20	0	7.50	5.40	14.00	11.80	6.50	6.50	− 1.693 (− 3.341–045)	0.044
0–1 year	0	0	33.30	0	0	0	0	33.30	0	33.30	0	0	0	0	0	0	0	0	− 0.667 (− 7.338–6.004)	0.845
2–5 years	0	4.80	0	4.80	4.80	9.50	4.80	19.00	19.00	9.50	0	0	4.80	4.80	9.50	4.80	0	0	− 3.109 (− 5.841–0.377)	0.026
6–11 years	0	0	0	0	1.80	1.80	12.70	5.50	12.70	9.10	3.60	0	10.90	5.50	14.50	10.90	7.30	3.60	− 1.231 (− 2.38–083)	0.036
≥ 12 years	0	0	0	0	0	0	0	0	0	0	0	0	0	7.10	21.40	28.60	14.30	28.60	0.104 (− 0.964–1.171)	0.849

*Serratia marcescens*	2002	2003	2004	2005	2006	2007	2008	2009	2010	2011	2012	2013	2014	2015	2016	2017	2018	2019	Change by year (95% CI)	*p*-value
	n = 1	n = 0	n = 1	n = 0	n = 2	n = 1	n = 2	n = 0	n = 1	n = 0	n = 0	n = 0	n = 2	n = 4	n = 3	n = 5	n = 3	n = 3		
Overall	3.60	0	3.60	0	7.10	3.60	7.10	0	3.60	0	0	0	7.10	14.30	10.70	17.90	10.70	10.70	− 1.281 (− 3.375–0.813)	0.231
0–1 year	14.30	0	14.30	0	28.60	0	14.30	0	14.30	0	0	0	0	0	0	14.30	0	0	N/A	N/A
2–5 years	0	0	0	0	0	20	0	0	0	0	0	0	0	20	0	20	40	0	0.942 (− 2.5–4.385)	0.592
6–11 years	0	0	0	0	0	0	12.50	0	0	0	0	0	12.50	12.50	12.50	12.50	12.50	25.00	0.566 (− 1.232–2.364)	0.537
≥ 12 years	0	0	0	0	0	0	0	0	0	0	0	0	12.50	25.00	25.00	25.00	0	12.50	− 0.859 (− 2.122–0.404)	0.182

Non-tuberculous mycobacteria	2002	2003	2004	2005	2006	2007	2008	2009	2010	2011	2012	2013	2014	2015	2016	2017	2018	2019	Change by year (95% CI)	*p*-value
	n = 0	n = 0	n = 0	n = 0	n = 0	n = 1	n = 0	n = 0	n = 0	n = 0	n = 0	n = 0	n = 1	n = 1	n = 3	n = 6	n = 6	n = 9		
Overall	0	0	0	0	0	3.70	0	0	0	0	0	0	3.70	3.70	11.10	22.20	22.20	33.30	1.591 (− 0.654–3.836)	0.165
0–1 year	0	0	0	0	0	0	0	0	0	0	0	0	0	0	0	0	0	0	N/A	N/A
2–5 years	0	0	0	0	0	50	0	0	0	0	0	0	0	0	0	50	0	0	0.771 (− 4.942–6.484)	0.791
6–11 years	0	0	0	0	0	0	0	0	0	0	0	0	9.10	0	9.10	18.20	27.30	36.40	1.562 (048–3.076)	0.043
≥ 12 years	0	0	0	0	0	0	0	0	0	0	0	0	0	7.10	14.30	21.40	21.40	35.70	0.331 (− 0.796–1.459)	0.565

*Burkholderia cepacia*	2002	2003	2004	2005	2006	2007	2008	2009	2010	2011	2012	2013	2014	2015	2016	2017	2018	2019	Change by year (95% CI)	*p*-value
	n = 0	n = 0	n = 0	n = 0	n = 0	n = 0	n = 0	n = 0	n = 0	n = 0	n = 1	n = 0	n = 0	n = 0	n = 1	n = 1	n = 2	n = 0		
Overall	0	0	0	0	0	0	0	0	0	0	20	0	0	0	20	20	40	0	0.568 (− 3.603–4.738)	0.79
0–1 year	0	0	0	0	0	0	0	0	0	0	0	0	0	0	0	0	0	0	N/A	N/A
2–5 years	0	0	0	0	0	0	0	0	0	0	0	0	0	0	0	0	0	0	N/A	N/A
6–11 years	0	0	0	0	0	0	0	0	0	0	33.30	0	0	0	33.30	33.30	0	0	− 0.46 (− 3.234–2.315)	0.745
≥ 12 years	0	0	0	0	0	0	0	0	0	0	0	0	0	0	0	0	100	0	0.446 (− 1.219–2.11)	0.6

*Achromobacter xylosoxidans*	2002	2003	2004	2005	2006	2007	2008	2009	2010	2011	2012	2013	2014	2015	2016	2017	2018	2019	Change by year (95% CI)	*p*-value
	n = 0	n = 0	n = 0	n = 0	n = 0	n = 0	n = 0	n = 0	n = 0	n = 1	n = 2	n = 0	n = 3	n = 3	n = 1	n = 0	n = 4	n = 1		
Overall	0	0	0	0	0	0	0	0	0	6.70	13.30	0	20	20	6.70	0	26.70	6.70	− 0.142 (− 3.242–2.958)	0.928
0–1 year	0	0	0	0	0	0	0	0	0	0	0	0	0	0	0	0	0	0	N/A	N/A
2–5 years	0	0	0	0	0	0	0	0	0	0	0	0	0	0	0	0	0	0	N/A	N/A
6–11 years	0	0	0	0	0	0	0	0	0	11.10	22.20	0	22.20	22.20	0	0	22.20	0	− 0.915 (− 2.833–1.003)	0.35
≥ 12 years	0	0	0	0	0	0	0	0	0	0	0	0	16.70	16.70	16.70	0	33.30	16.70	0.06 (− 1.453–1.572)	0.938

*Stenotrophomonas maltophilia*	2002	2003	2004	2005	2006	2007	2008	2009	2010	2011	2012	2013	2014	2015	2016	2017	2018	2019	Change by year (95% CI)	*p*-value
	n = 0	n = 0	n = 0	n = 0	n = 1	n = 0	n = 1	n = 4	n = 2	n = 1	n = 3	n = 3	n = 6	n = 9	n = 2	n = 8	n = 5	n = 9		
Overall	0	0	0	0	1.90	0	1.90	7.40	3.70	1.90	5.60	5.60	11.10	16.70	3.70	14.80	9.30	16.70	− 1.281 (− 3.375–0.813)	0.231
0–1 year	0	0	0	0	0	0	0	0	0	0	0	0	0	0	0	0	0	0	N/A	N/A
2–5 years	0	0	0	0	0	0	11.10	0	0	0	0	11.10	22.20	33.30	0	0	11.10	11.10	N/A	N/A
6–11 years	0	0	0	0	3.20	0	0	12.90	6.50	3.20	9.70	3.20	6.50	16.10	0	12.90	9.70	16.10	1.781 (− 1.375–2.813)	0.796
≥ 12 years	0	0	0	0	0	0	0	0	0	0	0	7.10	14.30	7.10	14.30	28.60	7.10	21.40	− 0.067 (− 2.452–4.897)	0.13

Figures are represented in percentages, indicating the proportion of the respiratory pathogen's presence within each age group over the different years. n = number of children or adolescents with a positive airway sample culture for the corresponding year. The change by year (95% CI) represents the coefficient obtained from regression modeling, indicating the percentage change per year, along with its corresponding 95% confidence interval and *p*-value. N/A = not applicable.

Throughout the entire study period, *S. aureus* (47.8%), *P. aeruginosa* (34.5%), and *A. fumigatus* (8.4%) exhibited the highest prevalence among respiratory pathogens. Together, these organisms constituted almost 95% of the overall prevalence over 18 years. In contrast, the least prevalent respiratory pathogens were NTM (0.72%), *B. cepacia* (0.69%), and *A. xylosoxidans* (0.48%) throughout the study period. Collectively, these organisms represented less than two percent of the overall prevalence over 18 years (Table 2).

**Table 2:** Prevalence rates of pathogens isolated according to consecutive years.

*Staphylococcus aureus*	2002 n = 94	2003 n = 89	2004 n = 102	2005 n = 130	2006 n = 138	2007 n = 134	2008 n = 141	2009 n = 162	2010 n = 161	2011 n = 171	2012 n = 157	2013 n = 105	2014 n = 152	2015 n = 153	2016 n = 154	2017 n = 132	2018 n = 130	2019 n = 133	Change by year (95% CI)	*p*-value
Overall	3.80	3.70	4.20	5.30	5.70	5.40	5.70	6.60	6.60	7.00	6.50	4.30	6.30	6.30	6.30	5.40	5.40	5.40	− 2.571 (− 3.502–1.64)	< 0.001
0–1 year	12.30	10.50	13.50	12.90	11.70	9.90	11.70	7.00	6.40	2.90	1.20	0	0	0	0	0	0	0	1.146 (− 1.955–4.247)	0.469
2–5 years	8.20	8.00	8.00	9.40	9.80	7.50	7.30	9.20	8.00	7.80	6.70	3.60	3.20	2.30	1.10	0	0	0	− 0.339 (− 1.5–0.822)	0.567
6–11 years	2.80	2.80	3.70	5.30	5.90	5.90	5.60	7.00	6.40	7.80	6.60	4.90	7.20	6.90	6.50	5.40	4.80	4.50	− 2.605 (− 3.55–1.659)	< 0.001
≥ 12 years	0	0	0	0.70	0.90	2.40	3.40	4.20	5.80	6.30	7.30	5.20	8.80	9.90	11.40	10.70	11.30	11.80	− 2.571 (− 3.502–1.64)	< 0.001

*Pseudomonas aeruginosa*	2002 n = 95	2003 n = 89	2004 n = 102	2005 n = 105	2006 n = 104	2007 n = 95	2008 n = 102	2009 n = 87	2010 n = 100	2011 n = 96	2012 n = 93	2013 n = 35	2014 n = 80	2015 n = 71	2016 n = 61	2017 n = 68	2018 n = 50	2019 n = 45	Change by year (95% CI)	*p*-value
Overall	6.30	6.00	6.90	7.20	7.10	6.30	6.90	5.90	6.80	6.40	6.30	2.40	5.40	4.80	4.10	4.60	3.40	3.10	− 2.985 (− 3.966–2.003)	< 0.001
0–1 year	16.50	13.70	14.10	12.00	10	8.80	9.20	6.00	4.80	2.80	1.20	0	0	0	0.40	0	0.40	0	0.811 (− 2.281–3.904)	0.607
2–5 years	7.50	8.60	9.40	9.90	10.30	8.40	8.10	7.10	7.10	7.30	7.10	3.40	3.40	1.90	0.60	0	0	0	− 0.513 (− 1.682–0.656)	0.39
6–11 years	3.70	3.10	5.10	5.50	4.60	4.80	5.70	5.50	9.20	7.50	7.30	3.30	7.70	7.30	6.80	5.90	3.70	3.30	− 2.828 (− 3.819–1.838)	< 0.001
≥ 12 years	0	0	0	1.30	3.40	3.40	4.70	4.40	4.40	6.40	8.10	1.30	9.80	9.80	8.40	13.80	10.80	10.10	− 2.985 (− 3.966–2.003)	< 0.001

*Haemophilus influenza*	2002 n = 8	2003 n = 10	2004 n = 7	2005 n = 17	2006 n = 36	2007 n = 28	2008 n = 44	2009 n = 40	2010 n = 33	2011 n = 34	2012 n = 26	2013 n = 8	2014 n = 46	2015 n = 34	2016 n = 27	2017 n = 21	2018 n = 12	2019 n = 11	Change by year (95% CI)	*p*-value
Overall	1.80	2.30	1.60	3.90	8.00	6.40	10	9.10	7.30	7.50	5.90	1.80	10.50	7.80	5.90	4.80	2.70	2.50	− 3.931 (− 4.95–2.912)	< 0.001
0–1 year	16.70	0	8.30	0	8.30	8.30	25.00	16.70	8.30	0	8.30	0	0	0	0	0	0	0	2.417 (− 1.02–5.854)	0.168
2–5 years	5.30	9.20	5.30	5.30	15.80	11.80	10.50	5.30	7.90	7.90	6.60	1.30	3.90	3.90	0	0	0	0	− 0.239 (− 1.625–1.146)	0.735
6–11 years	1.40	2.00	1.40	6.80	10.80	8.80	11.50	13.50	8.10	8.80	4.10	2.00	7.40	4.70	3.40	2.00	1.40	2.00	− 3.977 (− 5.132–2.821)	< 0.001
≥ 12 years	0	0	0	1.50	3.00	2.50	7.90	6.90	6.40	6.90	6.90	2.00	15.80	11.90	10.40	8.90	5.00	4.00	− 3.931 (− 4.95–2.912)	< 0.001

*Aspergillus fumigatus*	2002 n = 35	2003 n = 37	2004 n = 34	2005 n = 33	2006 n = 30	2007 n = 32	2008 n = 46	2009 n = 38	2010 n = 38	2011 n = 45	2012 n = 36	2013 n = 14	2014 n = 41	2015 n = 36	2016 n = 53	2017 n = 45	2018 n = 40	2019 n = 31	Change by year (95% CI)	*p*-value
Overall	5.30	5.60	5.00	5.00	4.50	4.80	6.90	5.70	5.70	6.60	5.40	2.10	6.20	5.40	8.00	6.80	6.00	4.70	− 1.002 (− 2.109–0.104)	0.076
0–1 year	10.70	18.70	20	12.00	8.00	9.30	6.70	8.00	4.00	1.30	1.30	0	0	0	0	0	0	0	0.6 (− 2.535–3.735)	0.708
2–5 years	11.60	10.50	7.20	9.90	9.40	8.30	11.00	5.50	6.10	6.60	5.50	1.70	2.80	2.20	1.70	0	0	0	− 0.895 (− 2.134–0.345)	0.157
6–11 years	2.00	1.40	1.70	2.00	2.40	3.40	6.80	6.10	7.10	8.80	7.80	3.40	8.80	7.80	10.20	8.80	6.10	5.10	− 1.231 (− 2.267–0.194)	0.02
≥ 12 years	0	0	0	0	0	0	0.90	3.60	2.70	4.50	1.80	0.90	8.90	8.00	17.90	17.00	19.60	14.30	− 1.002 (− 2.109–0.104)	0.076

*Serratia marcescens*	2002 n = 2	2003 n = 4	2004 n = 3	2005 n = 2	2006 n = 5	2007 n = 4	2008 n = 3	2009 n = 1	2010 n = 2	2011 n = 1	2012 n = 0	2013 n = 0	2014 n = 3	2015 n = 7	2016 n = 8	2017 n = 9	2018 n = 9	2019 n = 5	Change by year (95% CI)	*p*-value
Overall	2.90	5.90	4.40	2.90	7.40	5.90	4.40	1.50	2.90	1.50	0	0	4.40	10.30	11.80	13.20	13.20	7.40	− 1.35 (− 2.872–0.171)	0.082
0–1 year	0	0	0	0	100	0	0	0	0	0	0	0	0	0	0	0	0	0	N/A	N/A
2–5 years	6.30	25.00	12.50	12.50	6.30	12.50	6.30	6.30	6.30	6.30	0	0	0	0	0	0	0	0	− 2.325 (− 4.424–0.226)	0.03
6–11 years	5.00	0	5.00	0	5.00	5.00	5.00	0	0	0	0	0	10	25.00	20	5.00	10	5.00	− 0.346 (− 2.513–1.822)	0.754
≥ 12 years	0	0	0	0	6.50	3.20	3.20	0	3.20	0	0	0	3.20	6.50	12.90	25.80	22.60	12.90	− 1.35 (− 2.872–0.171)	0.082

Non-tuberculous mycobacteria	2002 n = 2	2003 n = 1	2004 n = 1	2005 n = 1	2006 n = 0	2007 n = 1	2008 n = 0	2009 n = 1	2010 n = 0	2011 n = 1	2012 n = 0	2013 n = 0	2014 n = 2	2015 n = 2	2016 n = 3	2017 n = 9	2018 n = 9	2019 n = 12	Change by year (95% CI)	*p*-value
Overall	4.40	2.20	2.20	2.20	0	2.20	0	2.20	0	2.20	0	0	4.40	4.40	6.70	20	20	26.70	1.131 (− 0.799–3.062)	0.251
0–1 year	50	50	0	0	0	0	0	0	0	0	0	0	0	0	0	0	0	0	− 2.167 (− 7.027–2.694)	0.382
2–5 years	25.00	0	25.00	0	0	0	0	0	0	0	0	0	25.00	0	25.00	0	0	0	1.05 (− 2.671–4.771)	0.58
6–11 years	0	0	0	4.30	0	4.30	0	4.30	0	4.30	0	0	4.30	8.70	4.30	21.70	21.70	21.70	2.093 (0.046–4.14)	0.045
≥ 12 years	0	0	0	0	0	0	0	0	0	0	0	0	0	0	6.30	25.00	25.00	43.80	1.131 (− 0.799–3.062)	0.251

*Burkholderia cepacia*	2002 n = 4	2003 n = 2	2004 n = 2	2005 n = 3	2006 n = 4	2007 n = 3	2008 n = 2	2009 n = 2	2010 n = 1	2011 n = 1	2012 n = 2	2013 n = 0	2014 n = 1	2015 n = 1	2016 n = 3	2017 n = 3	2018 n = 5	2019 n = 4	Change by year (95% CI)	*p*-value
Overall	9.30	4.70	4.70	7.00	9.30	7.00	4.70	4.70	2.30	2.30	4.70	0	2.30	2.30	7.00	7.00	11.60	9.30	0.969 (− 1.372–3.31)	0.417
0–1 year	25.00	25.00	0	12.50	12.50	12.50	0	12.50	0	0	0	0	0	0	0	0	0	0	− 0.042 (− 3.646–3.563)	0.982
2–5 years	12.50	0	12.50	12.50	18.80	12.50	12.50	0	6.30	6.30	6.30	0	0	0	0	0	0	0	− 1.512 (− 3.611–0.586)	0.158
6–11 years	0	0	0	0	0	0	0	11.10	0	0	11.10	0	11.10	11.10	22.20	11.10	11.10	11.10	1.465 (− 1.609–4.539)	0.35
≥ 12 years	0	0	0	0	0	0	0	0	0	0	0	0	0	0	10	20	40	30	0.969 (− 1.372–3.31)	0.417

*Achromobacter xylosoxidans*	2002 n = 0	2003 n = 0	2004 n = 0	2005 n = 0	2006 n = 0	2007 n = 0	2008 n = 0	2009 n = 2	2010 n = 1	2011 n = 2	2012 n = 5	2013 n = 1	2014 n = 4	2015 n = 5	2016 n = 6	2017 n = 1	2018 n = 9	2019 n = 6	Change by year (95% CI)	*p*-value
Overall	0	0	0	0	0	0	0	4.80	2.40	4.80	11.90	2.40	9.50	11.90	14.30	2.40	21.40	14.30	0.469 (− 2.718–3.656)	0.773
0–1 year	0	0	0	0	0	0	0	0	0	0	0	0	0	0	0	0	0	0	N/A	N/A
2–5 years	0	0	0	0	0	0	0	33.30	16.70	16.70	16.70	16.70	0	0	0	0	0	0	2.717 (− 0.389–5.823)	0.086
6–11 years	0	0	0	0	0	0	0	0	0	3.20	12.90	0	12.90	12.90	19.40	3.20	16.10	19.40	2.225 (0.404–4.047)	0.017
≥ 12 years	0	0	0	0	0	0	0	0	0	0	0	0	0	20	0	0	80	0	0.469 (− 2.718–3.656)	0.773

*Stenotrophomonas maltophilia*	2002 n = 4	2003 n = 3	2004 n = 5	2005 n = 5	2006 n = 6	2007 n = 6	2008 n = 9	2009 n = 10	2010 n = 11	2011 n = 4	2012 n = 10	2013 n = 4	2014 n = 18	2015 n = 20	2016 n = 16	2017 n = 22	2018 n = 25	2019 n = 20	Change by year (95% CI)	*p*-value
Overall	2.00	1.50	2.50	2.50	3.00	3.00	4.60	5.10	5.60	2.00	5.10	2.00	9.10	10.20	7.60	11.20	12.70	10.20	1.131 (− 0.799–3.062)	0.251
0–1 year	0	33.30	0	33.30	33.30	0	0	0	0	0	0	0	0	0	0	0	0	0	N/A	N/A
2–5 years	7.50	5.00	7.50	7.50	7.50	10	12.50	7.50	10	7.50	10	0	2.50	2.50	2.50	0	0	0	0.21 (− 2.71–3.706)	0.854
6–11 years	1.00	0	2.10	1.00	2.10	2.10	4.20	7.30	7.30	1.00	6.30	2.10	13.50	10.40	8.30	12.50	11.50	7.30	1.993 (0.046–2.14)	0.063
≥ 12 years	0	0	0	0	0	0	0	0	0	0	0	3.40	6.90	15.50	10.30	17.20	24.10	22.40	2.341 (− 0.469–2.982)	0.251

Figures are represented in percentages, indicating the proportion of the respiratory pathogen's presence within each age group over the different years. n = number of children or adolescents with a positive airway sample culture for the corresponding year. The change by year (95% CI) represents the coefficient obtained from regression modeling, indicating the percentage change per year, along with its corresponding 95% confidence interval and *p*-value. N/A = not applicable.

### Changes in age of first isolation of respiratory pathogens

The ages at which these pathogens were first isolated are as follows: *S. aureus* (3.35 ± 2.1 years), *H. influenza* (4.28 ± 2.7 years), *S. marcescens* (5.24 ± 4.09 years), *P. aeruginosa* (5.27 ± 2.9 years), *A. fumigatus* (7.31 ± 2.85 years). This is followed by *S. maltophilia* (8.95 ± 2.95 years), *B. cepacia* (9.055 ± 2.3 years), NTM (11.38 ± 2.06 years), *A. xylosoxidans* (11.71 ± 2.86 years).

**Figure 1:**
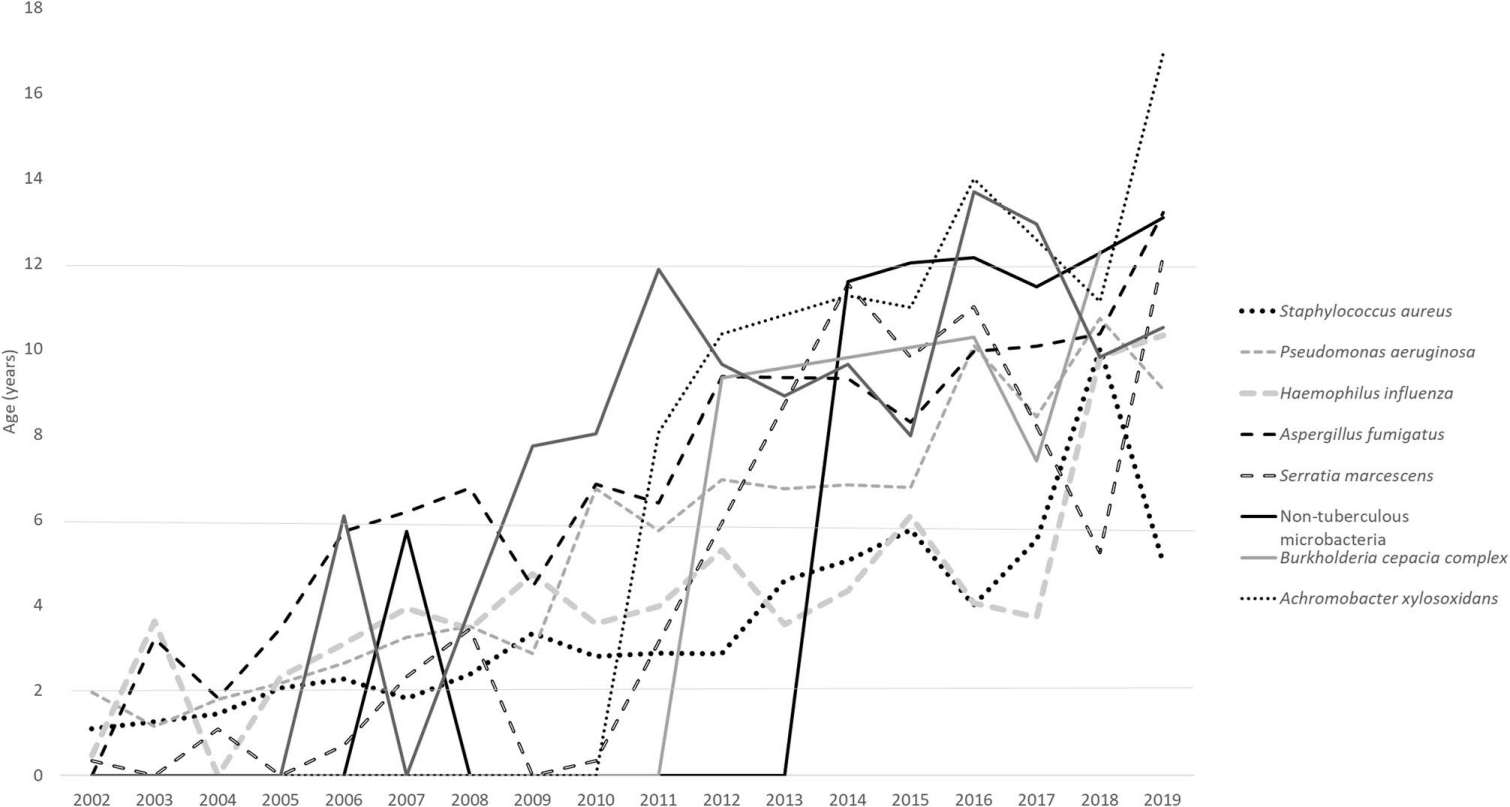
Mean age group of the first culture of CF organisms.

Over time, respiratory pathogens have shown an increase in the mean age of the first isolation: *S. aureus* (*p* < 0.001), *P. aeruginosa* (*p* < 0.001), *H. influenza* (*p* < 0.001), *S. marcescens* (*p* = 0.006), *A. Fumigatus* (*p* = 0.02), *B. cepacia* (*p* = 0.58), NTM (*p* = 0.052), *S. marcescens* (*p* = 0.308), *S. maltophilia* (*p* = 0.47), *A. xylosoxidans* (*p* = 0.80). The changes over years of these respiratory pathogens are illustrated in Fig. 1.

### Changes of overall and age-specific incidence and prevalence of CF organisms from 2002 to 2019

Amongst the organisms with the highest incidence, *S. aureus* showed the largest decline in incidence over time, followed by *H. influenza* and *P. aeruginosa*. Meanwhile, NTM and *B. cepacia* showed a non-significant increase in incidence. A similar pattern of change in prevalence was observed (Tables 1, 2).

With respect to age groups, incidence of *S. aureus, P. aeruginosa, H. influenza* and *A. fumigatus* in children < 2 years of age have remained unchanged. A similar pattern of change in prevalence was observed. Meanwhile, NTM showed a significant increase in both incidence and prevalence in children 6–11 years of age.

### Treatment

Throughout this study, a total of 29,203 medications (oral antimicrobials n = 18,367, 62.9%) were prescribed. The antibiotics that were increasingly prescribed include amikacin (3.09 ± 2.24 prescription/year, *p* = 0.003), amoxicillin/clavulanic acid (8.98 ± 2.17 prescriptions/year, *p* < 0.001), colistin (1.95 ± 0.3 prescriptions/year, *p* = 0.032), trimethoprim/sulfamethoxazole (18.1 ± 8.7, *p* < 0.001). Flucloxacillin (− 4.48 ± 1.073, *p* < 0.001), tobramycin (− 8.46 ± 4.7, *p* < 0.001) showed a decrease in prescription. Ciprofloxacin (− 6.049 ± 5.1 prescriptions/year, *p* = 0.068) and itraconazole (− 4.53 ± 1 prescriptions/year, *p* = 0.07) did not show any significant change over time.

Dornase alfa prescription increased by 16.74 ± 4.1 prescriptions/year (*p* < 0.001). The prescription of hypertonic saline nebulisation increased by 24 ± 4.6 prescriptions/year (*p* < 0.001). There were 7 children or adolescents on CFTR corrector or modulator therapy.

## Discussion

This paediatric-focused study evaluates annual changes in the incidence and prevalence rates of respiratory pathogens across different age groups, while also comparing medication prescription trends over an 18-year period. This study provides valuable data from a real-world clinical setting where infants under the age of one receive universal antimicrobial prophylaxis and, standardised respiratory pathogen surveillance is conducted by qualified personals using consistent sampling and microbiological testing protocols. In particular, obtaining samples through sputum and oropharyngeal suctioning is considered to have the highest concordance with BAL samples, rendering them more representative of lower airway infections compared to other sampling methods like throat or cough swabs^18^. The findings contribute to our understanding of the long-term trends in respiratory pathogens and associated clinical management in the paediatric population, particularly in the modern era of eradication therapy^15^.

Our study showed that together, *S. aureus* and *P. aeruginosa* make up the majority of respiratory pathogens both in terms of incidence (51.3%) and prevalence (82.3%). Data preceding 2000, report prevalence of these two respiratory pathogens to be higher at 95%^14^.

Registry data taken from 2018 to 2020 showed a prevalence of *P. aeruginosa* of 20.9%^17^ and *S. aureus* of 55.26% in children and adolescents under the age of 18. In comparison, our data shows a recent prevalence of *P. aeruginosa* of 17.6% and *S. aureus* of 45.3%. Of the less frequent respiratory pathogens, NTM prevalence was 4.3% from registry data vs 3.7% from our cohort and *B.cepacia* was 3.2% vs. 1.3% respectively.

In a recent publication by VanDevanter et al., a trend of decline in *P. aeruginosa* prevalence was observed, as evidenced by the examination and presentation of registry data within a comparable time frame^19^. Following this, Fischer et al. raised a crucial question regarding whether the observed changes in *P. aeruginosa* over time were also apparent in other respiratory pathogens of interest in CF^20^. We have demonstrated that over the past 18 years, the incidence and prevalence of the most common respiratory pathogens in CF such as *S. aureus*, *P. aeruginosa*, *H. influenzae* and *A. fumigatus* have decreased steadily. This significant decline of between 2 and 4% of individual respiratory pathogens are observed both in the incidence and prevalence. Meanwhile, less common organisms such as NTM*, B. cepacia* and *A. xylosoxidans, S. maltophilia* showed no significant change in terms of incidence and prevalence.

We also found that the incidence and prevalence of respiratory pathogens remain unchanged for infants up to 2 years of age across all respiratory pathogens. Additionally, we have found that our cohort of children and adolescents with CF are found to have a positive airway sample culture for these respiratory pathogens significantly later that the earlier years of this study.

Our centre has adopted the universal use of *S. aureus* prophylactic antibiotics in infants diagnosed with CF preceding this study period. In a systematic analysis performed which reviewed four studies, there was a weak indication that *P.aeruginosa* was isolated less frequently in children under three years and more frequently in children between three to six years in the prophylactic group^21^. In contrast, despite our universal use of prophylactic antibiotics in infants, our study shows (1) a decline in the incidence and prevalence of *P. aeruginosa*, (2) no  significant increase in the incidence and prevalence of organisms such as NTM and *B. cepacia* (3) an increase in the mean age of first isolation of respiratory pathogens of interest, (4) no change of incidence and prevalence of respiratory pathogen < 2 years of age. A contributing factor in terms of improvements in infection control practices may have helped keep our incidence and prevalence lower than the national average. While being potentially circumstantial, these findings suggest that the use of prophylactic anti-staphylococcal antibiotics is not associated with an increase in *P. aeruginosa* or increase in prevalence of other less common respiratory pathogen. Prospective studies such as the CF-START study in evaluating outcomes of prophylactic treatments will hopefully provide conclusive proof of its benefits and safety^21^.

By examining prescription trends, we have found that there is a rise in the use of anti-pseudomonal nebulised antibiotics such as amikacin and colistin. This suggests that *P.aeruginosa* is being more aggressively treated over time as both this antibiotics are considered as second line after tobramycin^22^. However, the increase in use of amikacin could also be attributed to an increase in NTM incidence and prevalence. Encouragingly, we have found that the emphasis on respiratory clearance has increased over time with the significant increase in the prescription of dornase alpha and hypertonic saline in our cohort.

Our study comes with certain limitations that warrant consideration. Firstly, the sputum and prescription data lack representation from external laboratories or pharmacies, potentially limiting the comprehensiveness of our findings. Additionally, we did not culture anaerobic bacteria and did not routinely test for co-infection with respiratory viruses, leading to an omission in addressing potential co-infections among these organisms in our study. Moreover, the annual frequency of NTM testing, as opposed to routine CF airway sample cultures, may result in an underrepresentation of NTM within our study cohort.

Thirdly, our data originated from a single CF centre in Australia, raising concerns about the generalisability of our findings to a broader population. Fourthly, our incidence calculation may involve a small number of children or adolescents intermittently found to have these respiratory pathogens in their airway samples. Finally, the relatively limited sample size of children and adolescents on CFTR modulators or correctors is noteworthy, as our study predates the widespread adoption that followed the approval and government funding of these medications in Australia. Current evidence suggests that while it may more difficult to obtain sputum samples in children on CFTR therapy, its’ impact on the growth of specific bacterial pathogens needs to be closely examined^23^. The low number of children or adolescents on CFTR modulators or correctors is an important aspect of this study as it will enable future comparison in a post-modulator era in the management of CF.

Our study has several strengths. First, we analysed a large number of sputum samples, both overall and in different age groups, providing a longitudinal comparison of changes in CF treatment over the past 18 years. This is the first study of such magnitude in children and adolescents with CF, providing age-specific incidence and prevalence, as well as prescription trends. In particular, our review of incidences of these organisms and the age of first positive culture provides additional information towards our understanding of CF respiratory pathogens over the past two decades.

Second, our study includes a large cohort of children born on or after January 1st, 2002, when newborn screening has already been well-established, allowing us to assess the acquisition of respiratory pathogens from shortly after birth over the past 18 years. Third, the practice of using prophylactic anti-staphylococcus antibiotics universally has given us the opportunity to assess the outcomes of its’ use over a significantly long period of time. While strong conclusions cannot be made without a non-prophylactic control arm, it does provide insight into the long-term impact of its’ implementation on respiratory pathogens in our cohort.

In summary, our study shows a change in the epidemiology of CF pathogens in a single large paediatric clinic that practices universal prophylaxis in children. First, we observed a decline in the incidence and prevalence of the most commonly found CF pathogens such as *S. aureus, P. aeruginosa, H. influenzae,* and *A. fumigatus*, as well as a delay in the first acquisition of these pathogens. However, less common pathogens such as *S. marcescens*, NTM, *B. cepacia, A. xylosoxidans*, and *S. maltophilia* did not show significant changes. Second, we found no change in the incidence or prevalence of respiratory pathogens in infants under 2 years of age over time.

## Data Availability

Data is available from the corresponding author, upon reasonable request.

## References

[1] Flume PA (2007). Cystic fibrosis pulmonary guidelines: Chronic medications for maintenance of lung health. Am. J. Respir. Crit. Care Med..

[2] Lyczak JB, Cannon CL, Pier GB (2002). Lung infections associated with cystic fibrosis. Clin. Microbiol. Rev..

[3] Pillarisetti N (2011). Infection, inflammation, and lung function decline in infants with cystic fibrosis. Am. J. Resp. Crit. Care Med..

[4] Spicuzza L (2009). Emerging pathogens in cystic fibrosis: Ten years of follow-up in a cohort of patients. Eur. J. Clin. Microbiol. Infect. Dis..

[5] Coburn B (2015). Lung microbiota across age and disease stage in cystic fibrosis. Sci. Rep..

[6] Abidin NZ (2021). Trends in nontuberculous mycobacteria infection in children and young people with cystic fibrosis. J. Cystic Fibrosis.

[7] Miall LS, McGinley NT, Brownlee KG, Conway SP (2001). Methicillin resistant *Staphylococcus*
*aureus* (MRSA) infection in cystic fibrosis. Arch. Dis. Childhood.

[8] Breuer O (2019). Changing prevalence of lower airway infections in young children with cystic fibrosis. Am. J. Resp. Crit. Care Med..

[9] Hilliard TN (2007). Bronchoscopy following diagnosis with cystic fibrosis. Arch. Dis. Childhood.

[10] Gilligan PH (1991). Microbiology of airway disease in patients with cystic fibrosis. Clin. Microbiol. Rev..

[11] Burns JL (1998). Microbiology of sputum from patients at cystic fibrosis centers in the United States. Clin. Infect. Dis..

[12] Govan J, Nelson J (1992). Microbiology of lung infection in cystic fibrosis. Br. Med. Bull..

[13] Millar F, Simmonds N, Hodson M (2009). Trends in pathogens colonising the respiratory tract of adult patients with cystic fibrosis, 1985–2005. J. Cystic Fibrosis.

[14] Razvi S (2009). Respiratory microbiology of patients with cystic fibrosis in the United States, 1995 to 2005. Chest.

[15] Ramsay KA (2017). The changing prevalence of pulmonary infection in adults with cystic fibrosis: A longitudinal analysis. J. Cystic Fibrosis Off. J. Eur. Cystic Fibrosis Soc..

[16] McKay KO, Waters DL, Gaskin KJ (2005). The influence of newborn screening for cystic fibrosis on pulmonary outcomes in New South Wales. J. Pediatrics.

[17] Ahern, S. *et al.* The Australian cystic fibrosis data registry: Annual report 2019 (2021).

[18] Schultz A, Caudri D (2018). Cough swabs less useful but induced sputum very useful in symptomatic older children with cystic fibrosis. Lancet Resp. Med..

[19] VanDevanter DR, LiPuma JJ, Konstan MW (2023). Longitudinal bacterial prevalence in cystic fibrosis airways: Fact and artifact. J. Cystic Fibrosis Off. J. Eur. Cystic Fibrosis Soc..

[20] Fischer AJ, Planet PJ (2023). A birth cohort approach to understanding cystic fibrosis lung infections. J. Cystic Fibrosis Off. J. Eur. Cystic Fibrosis Soc..

[21] Rosenfeld M, Rayner O, Smyth AR (2020). Prophylactic anti-staphylococcal antibiotics for cystic fibrosis. Cochrane Database System. Rev..

[22] Elborn J, Hodson M, Bertram C (2009). Implementation of European standards of care for cystic fibrosis—control and treatment of infection. J. Cystic fibrosis.

[23] Nichols DP (2021). PROMISE: Working with the CF community to understand emerging clinical and research needs for those treated with highly effective CFTR modulator therapy. J. Cystic Fibrosis.

